# A New Exposition of the Functions of the Nerves

**Published:** 1833-10

**Authors:** 


*A New Exposition of the Functions of the Nerves.* By JAMES WILLIAM EARLE. Part I. London, 1833. 8vo. pp. 195.

WE confess that at first we were somewhat angry with Mr. Earle for publishing his essay in this shape as a substantive book, and not sending it to us as a review of Dr. W. Philip's Treatise on the Vital Functions. Cut down a little, we said, it would have made a capital leader; but we must take it as it comes, and acknowledge that it is an honest, gentlemanly review, and obviously the production of a man of talent, who, while he impugns the soundness of Dr. Philip's conclusions, does homage to his genius. It cannot be denied, moreover, that, in spite of its having the air of an "answer," it contains much original matter. The following is Mr. Earle's division of the nerves:

"In unison with my plan of arranging the nerves according to their separate functions, I propose, that the term cerebral nerve, instead of as hitherto being applied to every one which passes through an aperture in the skull, should be restricted to the olfactory, ophthalmic and auditory nerves, on account of their being more particularly connected with the intellectual functions which are now generally regarded by physiologists as performed in some way or other by the brain, and from their being the most simple in their function, which is only that of transmitting impressions from their extremities to the brain, and from their filaments not being intermingled with those of any other nerve, that they should form the *first class*, and be considered as the only purely cerebral nerves.

"That the *second class* should comprehend every nerve by means of which muscles are subjected to the influence of volition; these are the anterior nerves, and belong to the anterior columns of the spinal marrow. As this division embraces the third, the fourth, the anterior root of the fifth, the sixth, the portio dura of the seventh, and the ninth nerves, it will be seen that I have partially adopted the classification of Sir C. Bell, and, consequently, consider all the medullary fibres below the first formation of the crura cerebri, as comprised under the term Anterior Columns of the spinal marrow, without regard to the circumstance of those fibres being encompassed at a particular part by other bands of medullary substance, termed pons varolii; or separated by other bodies which have been termed corpora olivaria.

"That the *third class* should comprehend every nerve belonging to the Posterior Columns of the spinal marrow; thus including the posterior root of the fifth, the glosso-pharyngeal, the pneumogastric and Spinal Accessory nerves, all of which, with the exception of the last, transmit impressions from their extremities to the brain, and have protuberances upon them just before their respective junctions with the posterior columns of the spinal marrow; by the term Posterior Columns is signified their whole extent as far as the termination of the restiform bodies in the posterior peduncles of the cerebellum. The ganglions on these posterior nerves will, in the course of these observations, always be distinguished from those of the sympathetic by the term posterior ganglions, for the sake of avoiding any misunderstanding.

" Lastly, that the *fourth class* should comprehend every nerve proceeding from the sympathetic ganglions.

" Every part to which branches of these nerves are distributed, whether its structure is muscular as that of the heart, or fibrous as that of the arteries and iris, has a power of motion altogether independent of any influence that the will can exercise.

" Such is the classification which I consider the distinct nature of the nerves obviously points out, and which I think is more particularly warranted by the well-known fact that nervous filaments however intermingled, whether united with others near to the brain and spinal marrow, or at a distance from them, never change their character. I must particularly beg the reader's attention to this arrangement, because it will enable him to understand more readily the observations I shall have to make respecting the functions of the nerves, and the effects resulting from their combination." (P.39.)

Mr. Earle supposes that a nervous fluid is constantly emanating from the brain, constituting the *one* vital power on which every function necessary to the continuance of life depends. The following passage, in which this theory is involved, is ingenious and well-written:

" There is but little evidence by which we may be guided in judging of the rapidity with which the nervous circulation is carried on, but we know that it is not so great as to prevent the voluntary muscles from becoming weary, as also that, when they are fatigued, they are required to be a considerable time at rest before their power is perfectly restored. The ordinary degree of rapidity may be, I think, inferred from observing what occurs in a limb whose nerves have been pressed upon some time, and which has become benumbed, or, as it has been more commonly called, asleep. When the pressure is removed, a sensation of tingling, vulgarly called pins and needles, is quickly perceived, and it is not until after the cessation of this sensation that the muscles are again completely qualified to act in obedience to the will. The same sort of tingling, though in a slighter degree, may frequently be perceived in the legs and feet after an unusually long walk, if the attention be directed to it; and also in the female breast during the secretion of the milk. The fact of our being sensible of the tingling distinctly proves the participation of the posterior nerves in the process by which the power which enables muscles to contract is restored; and, as this power cannot arrive at the muscles from the brain except along the anterior nerves, I consider this fact as affording strong evidence of the passage of the *cerebral influence* from the extremities of the anterior to those of the posterior nerves, and consequently it becomes a confirmation of the opinion that there is a circulation in the nerves. Although it cannot be precisely determined with what degree of rapidity this circulation is carried on, there is great reason to believe that it varies more or less in nearly every individual, of which the pulse is the index; for inasmuch as the circulation of the blood is dependent upon that of the nervous system, it must always bear a certain relation to the vigour and activity of the power by which it is supported." (P. 185.)

When Mr. Earle favours us with the second part of his investigations, we shall return to the subject, and give his theories at greater length.

